# Biological Activities and Molecular Docking of Brassinosteroids 24-Norcholane Type Analogs

**DOI:** 10.3390/ijms21051832

**Published:** 2020-03-06

**Authors:** Katy Díaz, Luis Espinoza, Rodrigo Carvajal, Marcos Conde-González, Vladimir Niebla, Andrés F. Olea, Yamilet Coll

**Affiliations:** 1 Departamento de Química, Universidad Técnica Federico Santa María, Avenida España 1680, Valparaíso 2340000, Chile; katy.diaz@usm.cl (K.D.); luis.espinozac@usm.cl (L.E.); rodrigo.carvajal@postgrado.usm.cl (R.C.); 2 Center for Natural Products Research, Faculty of Chemistry, University of Havana. Zapata y G, La Habana 10400, Cuba; mrconde@estudiantes.fq.uh.cu (M.C.-G.); vladyniem@gmail.com (V.N.); 3 Instituto de Ciencias Químicas Aplicadas, Facultad de Ingeniería, Universidad Autónoma de Chile, El Llano Subercaseaux 2801, Santiago 8900000, Chile

**Keywords:** biological activities, brassinosteroid analogs, mixture of analogs, molecular docking, BR1–BAK1 complex

## Abstract

The quest and design of new brassinosteroids analogs is a matter of current interest. Herein, the effect of short alkyl side chains and the configuration at C22 on the growth-promoting activity of a series of new brassinosteroid 24-norcholan-type analogs have been evaluated by the rice leaf inclination test using brassinolide as positive control. The highest activities were found for triol **3** with a C22(*S*) configuration and monobenzoylated derivatives. A docking study of these compounds into the active site of the Brassinosteroid Insensitive 1(BRI1)–ligand–BRI1-Associated Receptor Kinase 1 (BAK1) complex was performed using AutoDock Vina, and protein–ligand contacts were analyzed using LigPlot^+^. The results suggest that the hydrophobic interactions of ligands with the receptor BRI1^LRR^ and hydrogen bonding with BAK1 in the complex are important for ligand recognition. For monobenzoylated derivatives, the absence of the hydrophobic end in the alkyl chain seems to be compensated by the benzoyl group. Thus, it would be interesting to determine if this result depends on the nature of the substituent group. Finally, mixtures of *S*/*R* triols **3**/**4** exhibit activities that are comparable or even better than those found for brassinolide. Thus, these compounds are potential candidates for application in agriculture to improve the growth and yield of plants against various types of biotic and abiotic stress.

## 1. Introduction

Brassinosteroids (BRs) are a large group of polyhydroxylated sterol derivatives found in the plant kingdom in extremely low amounts and eliciting very important functions, such as plant growth regulation [1,2] and cell division and differentiation in young tissues of growing plants [3,4,5]. As a result of these features, BRs were recognized as the sixth class of plant hormones [6,7].

Their natural occurrence, biological activities, and structure–activity relationships (SAR) have been reviewed by different groups [6,8,9,10] and brassinolide (BL, **1**) and castasterone (CS, **2**) are the most active and widely distributed. The low amount of BRs produced in plants has prompted the quest for synthetic BRs derivatives, and so far, 137 analogs have already been reported [6]. However, structural variations arising from the type and position of functions in the A, B rings, fusion A/B ring, and alky side chain should be considered in the design of new BRs analogs. This means that the knowledge of functional groups responsible for biological activity is of paramount importance for a successful synthetic strategy. General requirements for BRs’ growth-promoting activity have been obtained by structure–activity relationships (SAR) studies using mainly two different protocols to quantify the bioactivity of new BRs analogs, namely, Bean Second Internode Bioassay (BSIB) and Rice Lamina Inclination Test (RLIT) assay [6,11,12].

Briefly, these studies have established that for high biological activity, the following factors are required: the presence of 2α-OH and 3α-OH in ring A; the absence or change in the configuration of both hydroxyl groups bringing about a decrease in activity following the order 2α, 3α dihydroxy > 3α hydroxy > 3 dehydro > 3β hydroxy > 2,3-didesoxy [13]; the 7-oxa-lactone group plays a more important role in the plant growth-promoting activity than the 6-oxo group, and therefore transforming the 7-oxa-6-oxolactone to 6-oxa-7-oxolactone dramatically reduces its activity [13,14,15,16]; the epimerization of BL at C5 leads to 5-epi-brassinolide, which is almost completely inactive in the RLIT assay [17]; most natural bioactive BRs possess a side alkyl chain carrying a vicinal 22*R*, 23*R* diol structural functionality that also determines the activity—however, Takatsuto et al. have shown that the configuration of an alkyl group at C24 becomes more important than the configuration of the vicinal diol [12,18].

Based on these results, different approaches have been developed to obtain synthetic BRs analogs with a series of structural variations while keeping those functional groups considered essential for bioactivity. One of these has been focused on the introduction of structural changes in the side alkyl chain but keeping common patterns of organic functions in the A/B rings and *cis-trans* fusion between them. In this way, BRs analogs with shorter side chains [19,20,21,22] and/or including a variety of substituents [23,24,25,26,27,28,29,30,31,32,33,34,35,36,37,38,39] have been reported. The biological activity of these synthetic BRs analogs seems to depend on the spatial distribution of oxygen atoms instead of the presence or absence of one specific functional group in the molecule [11,18], and consequently some new SAR have been proposed [40,41,42]. 

**Figure 1:**
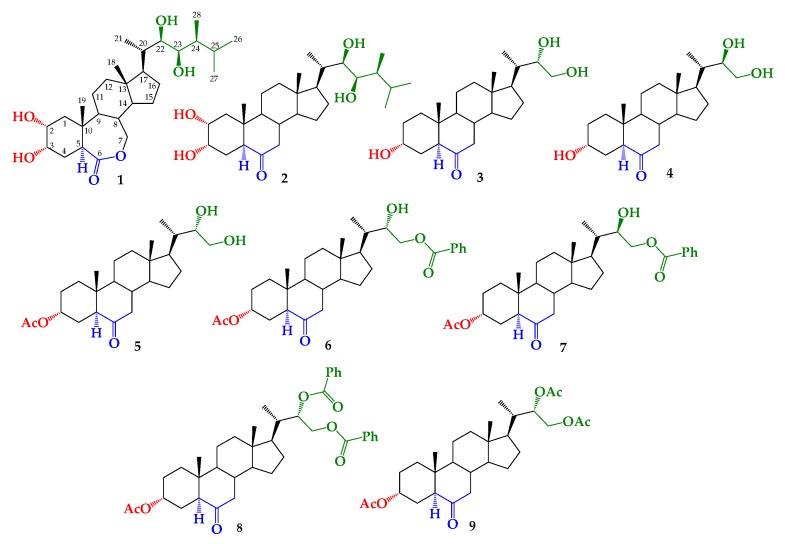
Structure of brassinolide (BL) (**1**), castasterone (CS) (**2**) and synthetic brassinosteroids (BRs) 24-nor-5α-cholan type analogs **3**–**9**.

To determine the structural effect of the side alkyl chain on growth-promotion activity, we have previously reported the synthesis of BRs 24-nor-5α-cholan type analogs (compounds **3**–**9**, Figure 1) [43,44]. These compounds were obtained by the dihydroxylation of a terminal olefin synthesized from hyodeoxycholic acid [43,44]. Thus, the aim of this study is to evaluate the growth-promoting activity of these analogs by using the RLIT and to explain the observed SAR by applying molecular docking.

In addition, unresolved epimeric mixtures (**3**/**4**, 1.0:0.24; **3**/**4**, 1.0:0.9) obtained as raw products from the synthetic reactions were also assayed.

## 2. Results and Discussion

### 2.1. Chemistry

Brassinosteroids 24-nor-5α-cholan type analogs have an alkyl side chain shorter than that of BL (see Figure 1). These analogs have been previously synthesized by the dihydroxylation of a terminal olefin (3α-acetoxy-24-nor-5α-cholan-22-en-6-one) obtained from hyodeoxycholic acid in a six-step route [43]. Briefly, the formation of vicinal diol function in the alkyl chain was performed by direct Upjohn dihydroxylation or direct Sharpless asymmetric dihydroxylation [44]. Application of these transformations led to mixtures of 22*S*/22*R* epimers of 22,23-dihydroxy-6-oxo-24-nor-5α-cholan-3α-yl acetate, with different diastereomeric ratios, i.e., 1.0:0.24 and 1.0:0.9, respectively [43,44]. Compound **5** was obtained by recrystallization from the former mixture [43]. Saponification reaction (K_2_CO_3_/CH_3_OH, reflux) of these mixtures leads to mixtures of **3**/**4** with the same epimer proportion, and the benzoylation reaction produced a mixture of compounds **6**–**8**. Finally, analogs **3** and **4** were obtained by the saponification of monobenzoylated derivatives **6** and **7** [44]. 

### 2.2. Bioactivity in the Rice Lamina Inclination Assay of Brassinosteroids Analogs.

**Figure 2:**
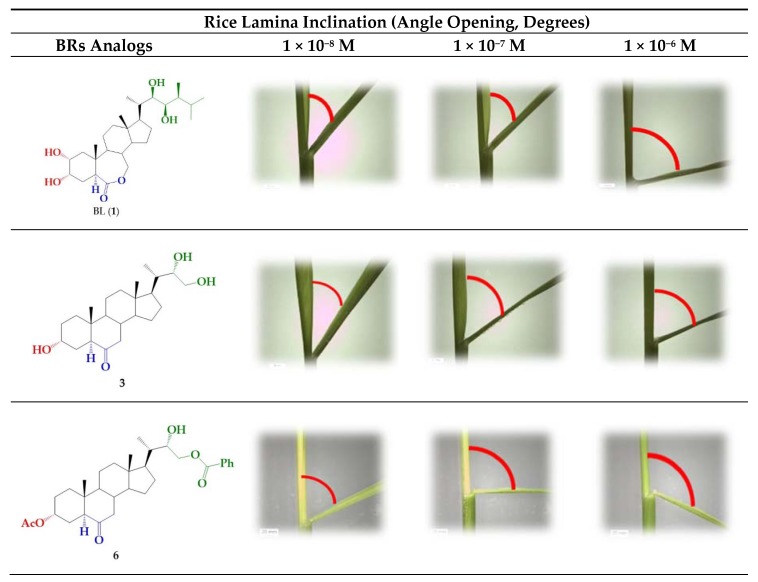

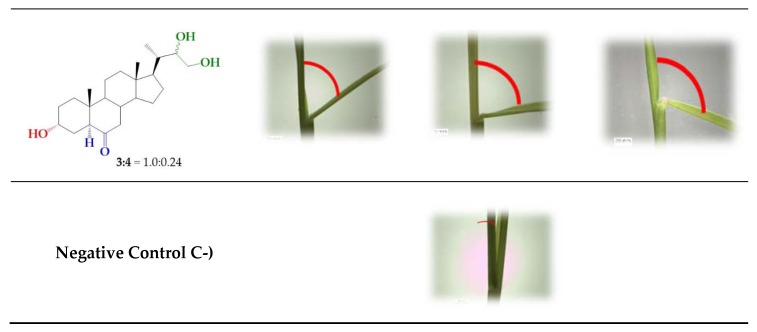
Rice-lamina assays using the second leaf lamina joints of excised leaf segments treated with BR analogs (**3** and **6**) and the epimeric mixture **3**/**4** (1.0:0.24) at different concentrations: 1 × 10^−8^, 1 × 10^−7^, and 1 × 10^−6^ M. BL (**1**) was used as positive control at the same concentrations.

A number of bioassays, such as first bean internode, root growth, and rice lamina inclination have been developed to evaluate the growth-promoting activity of BR derivatives [6,37,45]. In this work, the activity of BR 24-norcholane-type analogs was evaluated using the rice lamina inclination test because of its specificity and high sensitivity for BL and related compounds [37,45,46]. Figure 2 shows some typical results obtained for BL, which were used as positive control, and the more active BRs analog (**3** and **6**) and the unresolved epimeric mixture (**3**/**4**).

The bending angles were measured as the difference between the induced angle produced by treatment with each compound and that found for the negative control. 

The results obtained for BL, analogs **3**–**5**, **6**–**9**, and unresolved mixtures with different proportions of **3**/**4** are shown in Table 1. These values were obtained by averaging the results of two independent experiments with at least six replicates each (*n* = 12).

Interestingly, this data clearly indicate that 24-nor-5α-cholan-type analogs exhibit a considerable growth-promoting activity, as shown by the increased bending angle in rice seedlings. Even more, in the cases of analogs **3**, **6**, and **7** and mixtures of **3**/**4**, the measured activities in the whole range of tested concentrations are similar to those exhibited by BL. In the range of 1 × 10^−6^ to 1 × 10^−7^ the BRs analog with *S* configuration at C22 (**3**), it is three times more active than the *R* epimer (**4**). However, this difference disappears when a benzoyl group is attached to the side chain, i.e., compounds **6** and **7**. The calculated bending values for mixtures **3**/**4** can be obtained by using the individual activities obtained for analogs **3** and **4** and the epimeric composition determined by NMR, i.e., 1.0:0.24 and 1.0:0.9 [44]. The calculated bending angles at 1 × 10^−7^ M are 54 ± 4 and 46 ± 4, respectively, which compare quite well with the experimental values. Thus, these results suggest that the activity of mixtures of **3**/**4** comes from independent contributions of both diasteroisomers. From a practical point of view, the use of mixtures instead of pure epimers implies important savings in cost and synthetic efforts, since the steps of separation and purification would not be necessary, and the reaction product could be used directly. In this context, the total reaction yield becomes the main parameter to consider when choosing a synthetic strategy to obtain this type of analogs and, therefore, the Sharpless method is the more convenient route (95% yield) [44].

These results suggest that 24-norcholane-type analogs are active and worth exploring the effect of chemical structure on growth-promoting activity.

**Figure 3:**
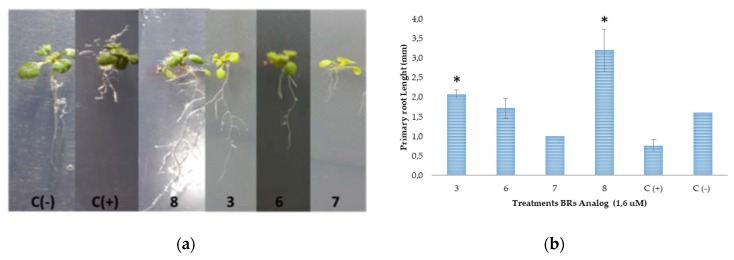
Influence of BR analogs (**3**, **6**, **7**, **8** and BL (C+)) on the root elongation of *A. thaliana*. (**a**) Representative pictures of primary root growth of 15-day-old wild-type plants grown at 21 °C and incubated in growth chambers under conditions 16 h light/8 h dark, 90 μE m^−2^ s^−1^; (**b**) Bar plot of primary root length (mean ± s.d., *n* = 15); (*) Indicate statistically significant differences between positive control (BL) and analogs treatments at the 0.05 significance level (Student’s *t*-test).

To simplify the data analysis, the only results we will consider are those obtained at 1 × 10^−7^ M for correlation between chemical structure and biological activity. The results indicate that the activity of 22*S*-epimer **3** is twice that shown by the 22*R*-epimer **4**, and it is much higher than the activity exhibited by analog **5**, which corresponds to the *S* epimer with the C3 hydroxyl group functionalized by an acetyl group. Thus, these results seem to indicate that both the C22 configuration and the presence of hydroxyl groups in these two specific positions are essential for biological activity. However, no activity differences are observed in the epimeric couple **6** and **7** where the hydroxyl groups at C3 and C23 have been transformed to acetyl and benzoyl groups, respectively. Additionally, both **6** and **7** produce bending angles similar or even higher than that obtained for 22*S*-epimer **3**. In other words, the analogs with a complete substitution of hydroxyl groups by acetyl and/or benzoyl groups keep a considerable activity as compared with epimer **3**, independently of C22 configuration. To make sure that these results are independent of the assay used to determine activities, we have also evaluated the effect of the most active compounds on the root elongation of *Arabidopsis thaliana* by measuring the primary root length of wild-type plants of *A. thaliana*. Representative pictures and a bar plot of the experimental lengths are shown in Figure 3.

Interestingly, the results indicate that the effect on root growth is strongly dependent on the chemical structure of the applied BRs analog (Figure 3a,b). The effects of BRs on root development using *Arabidopsis thaliana* as a model have been recently reviewed [47] and is generally accepted that BRs impact on meristematic cell proliferation and cell elongation through an autonomous stimulatory effect [48,49,50,51,52]. At 1 uM concentration, BL and **7** inhibit the root elongation in almost 50% of negative control (Figure 3b). These results are in line with previous reports indicating that BRs’ effects on the elongation of primary roots depend on the used concentration; i.e., BRs stimulate root growth at very low concentration, whereas an inhibitory effect is observed when the concentration exceeds a threshold value (1–100 nM BL) [48,50,53]. High concentrations of BL induce cell elongation in the meristem and reduction of the number and length of meristematic cells [50,52]. In this context, our results indicate that BL and compound **7** produce similar inhibitory effects, and therefore, they must act by identical mechanisms leading to the same bioactivities. On the other hand, compound **8** almost duplicates the root length of the negative control and induces lateral root formation (Figure 3a). Thus, the root growth enhancement of compounds **3** and **8** can be explained as the result of an alternative mechanism of action [53], which gives different bioactivities in *Arabidopsis* as compared to BL. Considering that the inhibitory effect of **7** is not much larger than that observed for **6** (values of root length are not significant different from BL, Figure 3b), it can be concluded that both epimers, C22(*S*) and C22(*R*), have a similar effect on primary root elongation. Therefore, our results suggest that 24-nor-5α-cholan-type analogs exhibit activity on *A. thaliana* as well, and that this effect depends on the chemical structure following a similar pattern in both assays.

In summary, in both bioassays, benzoylated derivatives **6** and **7** mimic BL, while dibenzoylated compound **8** acts in a completely different way. Finally, BRs analog **3** and BL behaves in identical and opposite ways in the RLIT and root elongation bioassays, respectively.

These results are in line with the proposal of some authors that the growth-promoting activity of BRs analogs depends mainly on the spatial distribution of oxygen atoms or the conformations that the molecule could adopt instead of the presence or absence of one specific functional group [40,54]. Thus, to get a better understanding of these structural effects, a molecular docking study was carried out.

### 2.3. Molecular Docking

Molecular docking is a computational tool that attempts to predict the non-covalent interactions between macromolecules or between small ligands and a macromolecule, starting from their unbound conformations [55]. On the other hand, it is well established that brassinosteroid perception occurs in a two-step mechanism [56,57], namely, the binding of BL to a hydrophobic surface groove on receptor BRI1^LRR^ domain [58] and the induced heterodimerization of BRI1^LRR^ with BAK1/SERK1 LRR. Formation of this ternary complex results in fully BRI1 activation [59]. To gain further insights into the way that the activity of BRs 24-nor-5α-cholanic type analogs is determined by the molecular structure, we performed the rigid docking of these compounds into the active site of the BRI1–BAK1 complex (PDB: 4m7e) using AutoDock Vina [55]. To determine the effect of C22 configuration and the presence of hydroxyl groups both in C3 and C23, analogs **3**, **4**, **6**, **7**, and **8** were chosen for this docking study.

**Figure 4:**
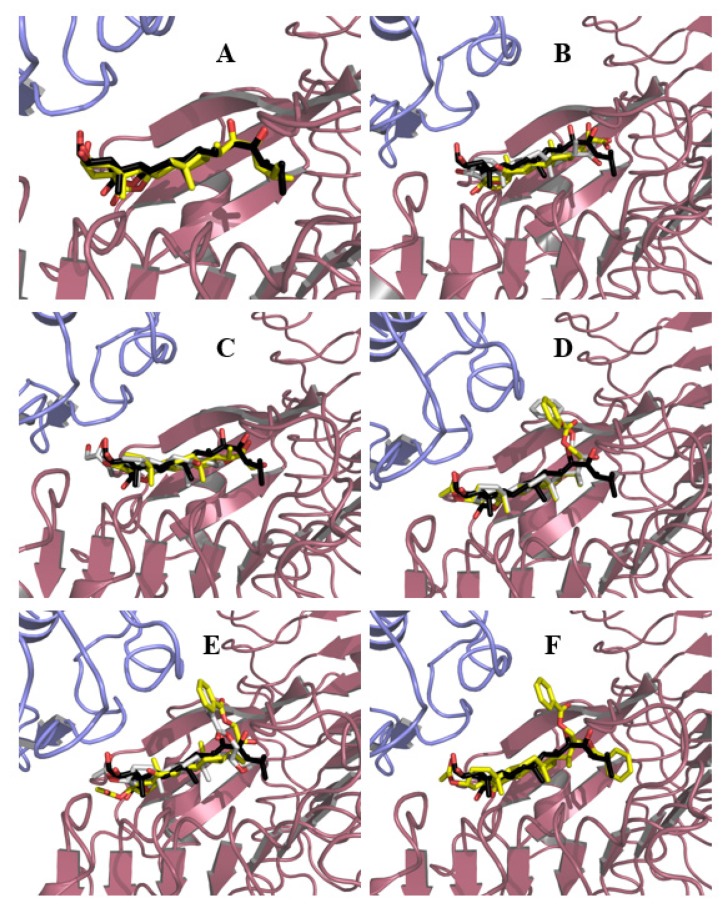
Binding modes of BL and synthetic ligands into BRI1–BAK1 heterodimer. The BL crystal pose is shown in black and red sticks | BRI1 and BAK1 are represented as pink and blue cartoons, respectively. Binding modes #1 and #2 for ligands are represented as yellow and white sticks, respectively. (**A**) BL (**1**, one binding mode); (**B**) Compound **3** (two binding modes); (**C**) Compound **4** (two binding modes); (**D**) Compound **6** (two binding modes); (**E**) Compound **7** (two binding modes); (**F**) Compound **8** (one binding mode).

In a first step, the natural ligand, BL, was redocked in order to assess the quality of the search parameters. Although two poses for BL were obtained, differing only in the orientation of the hydrophobic side chain, it was found that the one with the lowest energy is very similar to the crystal structure pose (Figure 4A). This result indicates that the search parameters were correct and consequently suitable to use for docking the synthetic analogs in the BRI1–BAK1 complex. The results of the pose analysis are shown in Table S1.

Since an approximately equal number of poses and similar energies were obtained for compounds **3**, **4**, **6**, and **7**, both conformations were kept for further analysis. In the case of compound **8**, multiple conformations were obtained, and only the most populated cluster of solutions was considered for analysis. Figure 4 shows the different binding modes obtained for BL and analogs **3**, **4**, **6**–**8.**

From this figure, it can be seen that all compounds, except analog **3**, adopt an orientation similar to that of BL (Figure 4A), i.e., the hydrophobic face of the steroid is oriented toward the hydrophobic LRR sheets, at least in one of their binding modes (Figure 4B,C,E,F). However, for compound 3 (Figure 4D), it is observed that the steroid core is completely flipped. This behavior is likely the result of subtle differences between both faces of the steroid and the absence of the hydrophobic end on the alkyl side chain due to the truncated nature of the derivative. In the case of compound **6**, this truncation is partially mended by the introduction of a benzoyl group on C23 (Figure 4B). At first sight, it seems reasonable to assume that the phenyl ring can be accommodated in the same cavity where the end into which the BL side alkyl chain fits, but this was not observed at any appreciable extent in the poses (only two poses out of the 30 presented fit this description), highlighting that further studies are needed in order to better understand the nature of the interactions of this analog. For compound **8**, one of its benzoyl groups (C23) is introduced in the cavity (Figure 4C), but in this case, this might be attributed to the presence of a second benzoyl group attached to C22 that forces the one on C23 to fit into the cavity, this being the only “acceptable” pose. It could be theorized that precisely this ring protruding out of the binding site might be responsible for the lesser activity of compound **8** as compared to compound **6**. It is imperative to keep in mind that BRs analogs are being docked into the formed heterodimer when actually the steroid binding is what drives its formation. Thus, it could be possible that steric hindrance with Brassinosteroid Insensitive 1 (BRI1) residues may disfavor the binding of BRI1-Associated Receptor Kinase 1 (BAK1) and hence block the complete activation of the receptor. In the case of the C22 isomers of compounds **3** and **6**, the poses do not differ much from those described so far for their counterparts, except the second binding pose of compound **3**, in which the side chain orients toward BAK1, differs from what is observed for BL in the crystal structure.

To better explain the difference in the activity of the synthesized analogs, the protein–ligand contacts were also analyzed. These contacts were visualized using LigPlot^+^ [60,61] (see Figures S1 and S2 and Table S2 Supplementary Material).

The results indicate that hydrophobic interactions are very similar for all compounds; all of them interact favorably with residues Tyr597 and Trp564, which are considered important for specific ligand recognition [62]. The biggest differences are encountered in the hydrogen bond networks. Most of the hydrogen bonds between compound **1** and the BRI1–BAK1 complex are absent in the complexes formed with the synthetic analogs. However, for compounds exhibiting the highest activity in this study, the hydrogen bond between the amino group of the main chain of Ser647 and oxygenated moiety attached to either C22 or C23 is still conserved. This interaction proved to be stable and strong in molecular dynamics simulations carried out on BL and synthetic ligand–BRI1–BAK1 complexes [63] and is absent in compound **8**. This absence can also contribute to explain the reduced activity of this compound.

However, an interesting situation is encountered for compound **4**, which is the C22(*S*) stereoisomer of compound **3**. This compound is the only one that interacts via hydrogen bonds with residues His61 and Val62 of BAK1. These interactions are also observed in a brassinolide–BRI1–BAK1 crystal structure [56] and are deemed very important for its recognition; this is achieved by flipping the orientation of the side chain, which is thought to be favored by the presence of the diol system in a configuration similar to that of BL. However, the activity of this compound is moderate, which proves the importance of the hydrophobic end of the side chain in the recognition process and correct activation of the receptor [32].

Evidently, further knowledge on the molecular factors determining the activity of the synthesized compounds can only be attained with molecular dynamics simulations. Moreover, there are several receptors that recognize BL in *Arabidopsis thaliana* alone [56], with key variations in the amino acid sequence that can tilt the scales on recognition and rejection, so the problem of correlating the activity of a compound with a specific receptor is further aggravated. Despite these limitations, our results suggest that the alkyl side chain in BL contributes significantly to recognition through the interactions of the hydrophobic end with BAK1. For the BR analogs studied in this work that carry the shortest side chain, the absence of this group might be compensated by the presence of a benzoyl group. Thus, it is interesting to determine if this result obeys a general behavior or if it depends on the nature of the substituent group.

## 3. Materials and Methods 

### 3.1. Biological Activity

#### 3.1.1. Rice Lamina Inclination Test

The biological activity of the compounds was evaluated by the rice lamina inclination test [64,65], according to a previously described procedure [37] and using a Zafiro cultivar (*Oryza sativa*) provided by the Institute of Agricultural Research (INIA-Quilamapu-Chile). After soaking rice seeds in sterile distilled water for 24 h, the seeds were selected and cultivated at 22 °C in a plant growing chamber under a photoperiod of light (16 h)/darkness (8 h) in pots with soil and plenty of water. Uniform seedlings presenting the second internode of the rice blade were selected for cutting. These segments were deposited in sterile distilled water in Petri dishes for 24 h. Subsequently, six segments per treatment were incubated in Petri dishes containing distilled water (60 mL), and the amount of test compound needed to reach final concentrations equal to 1 × 10^−8^ M, 1 × 10^−7^ M, and 1 × 10^−6^ M. BR analogs 3–8, mixtures 3/4, and BL were all added in aqueous solution with 0.001% ethanol, whereas only sterile distilled water was added for the negative control. Each experiment was performed in duplicate. Finally, after incubating for 48 h at 25 °C in darkness, the angles developed between the blade and the sheath were measured. Images were taken using a Leica EZ4HD Stereo Microscope with camera software. Data from at least 12 independent repeats were used to determine the significant differences between the positive control and treatments. Mean values with at least significant difference (*p* < 0.05; Student’s *t* test) were considered.

#### 3.1.2. Root Elongation of A. Thaliana

The effect of analogs **3**, **6**, **7**, and **8** on root growth was evaluated using ecotype Col-0 of *A. thaliana* (obtained from Arabidopsis Biological Resource Center). Sterilized seeds were incubated in Petri plates containing Murashige and Skoog media, vitamins, 1% sucrose; then, these were solidified with 1% agar. Previously to germination, seeds were stratified in cold (4 °C) and darkness for three days. Then, each tested compound was added, dissolved in water with 0.001% ethanol, reaching a 1.6 μM concentration in the plate. BL at the same concentration and pure solvent were used as positive and negative controls, respectively. Plates were incubated in growth chambers with photoperiods of 16 h light/8 h dark, 90 μE m^−2^ s^−1^. After seven days of germination, the measurement of primary root length was performed with a ruler using 15 plants by treatment and controls. Experiments were performed in triplicate. Data from at least 15 independent repeats were used to determine the significant differences between the positive control and treatments. Mean values with the least significant difference were considered (*p* < 0.05; Student’s *t* test).

### 3.2. Molecular Docking

#### 3.2.1. Ligand/Molecular Target Selection and Preparation

Two-dimensional structures of the steroids for the study were obtained using ChemDraw Professional 15.0 (Perkin Elmer, Waltham, MA, USA). Three-dimensional structure coordinates were generated and preliminarily optimized with MM2 force-field–Steepest Descent Algorithm [66] implemented in Chem3D 15.0 (Perkin Elmer). All structures were further optimized through the PM7 semi-empirical method implemented in MOPAC 2016 code (http://OpenMOPAC.net). All ligands were searched in the Protein Data Bank (PDB) and the obtained PDB-files were converted into PDBQT format (input for AutoDock Vina) [55]. The charges on the ligand atoms were generated using the Gasteiger model, nonpolar hydrogens were merged, and default rotating bonds were retained using the TORSDOF utility in AutoDockTools3 (http://mgltools.scripps.edu) [67].

The crystal structure of the protein (Brassinosteroid Insensitive 1 (BRI1) in complex with BAK1 (BRI1-Associated Receptor Kinase 1) and the natural ligand (BL), PDB ID: 4m7e) resolved at 3.60Å was retrieved from the Protein Data Bank (http://www.rcsb.org). The structure was optimized using pdb2pqr.py (Version 2.0.0) implemented in the web server PDB2PQR (http://nbcr-222.ucsd.edu/pdb2pqr_2.0.0/), using the AMBER force field, and the protonation state of ionizable groups at pH 8 was assigned using PROPKA 4.5 [68,69]. The grid search size was selected as 22 × 22 × 22 Å3 [67] with the center of the simulation box matching the center of the natural ligand, BL.

#### 3.2.2. Docking Procedure and Analysis of Protein–Ligand Complexes

Docking simulations were performed using Auto Dock Vina 1.1.2. The active site of the proteins heterodimer was kept rigid, and non-flexible docking was carried out. The docking parameters were set to default except for the following: exhaustiveness = 32 and num_modes = 2. The Vina code predicts the adopted conformations with the binding affinity (kcal/mol). The best two docked conformations were analyzed according to the binding affinity obtained from 15 independent runs to produce 30 final docked poses. These poses were clustered according to the Root-Mean-Square-Deviation (RMSD), with a cut-off of 1.5 Å among poses. The binding energy of the cluster is the average binding energy of all the conformations present in it. The cluster with the lowest binding energy and highest number of conformations was selected as the representative binding mode of that particular ligand.

Graphical representations of the protein–ligand complexes were prepared using PyMol^TM^ Version 2.1.0 (Schrödinger, New York, NY, USA, https://pymol.org/).

## 4. Conclusions

Seven new brassinosteroids analogs with a 24-norcholane-type of side chain, different configurations at C22, and their mixtures were tested for bioactivity using the Rice Lamina Inclination Test and root elongation in *A. thaliana*. Even though these two assays gave different activity results, both indicate that short-chain analogs with slightly bulky substituents in the chain have plant growth-promoting activities comparable to natural brassinosteroids. The activity of *S* epimer (**3**) is higher than that shown by the *R* epimer (**4**), but for compounds with a benzoyl group in the side chain, the activities become similar (**6** and **7**). The results from a molecular docking study suggest that this behavior could be explained in terms of hydrophobic interactions of the ligand with the receptor BRI1^LRR^ and after formation of the complex BRI1–Ligand–BAK1 hydrogen bonding of the ligand with BAK1. Thus, BR analogs with shortest side chain might be as effective as natural BRs as long as the chain is substituted with a hydrophobic group. It is noteworthy that mixtures of *S* and *R* stereoisomers (**3**/**4**) also exhibit excellent activities resulting from the independent contributions of each epimer. Thus, both BR analogs with 24-norcholane type side alkyl chains (**3** and **4**) and their epimeric mixtures (**3**/**4**) could be good candidates for a possible application in agriculture to improve the growth and yield of plants against various types of biotic and abiotic stresses. From the point of view of cost and time, the use of active mixtures instead of pure compounds could allow overcoming the economic constraints associated to chemical synthesis, which are currently the main limitations for using large-scale BRs in the fields.

## Figures and Tables

**Table 1:** Effect of BR 24-norcholane type analogs and epimeric mixtures of **3/4** on lamina inclination of rice seedlings. Brassinolide was used as positive control.

Bending Angle Between Laminae and Sheaths (Degrees ± Standard Error)			
Compounds	1 × 10^−8^ M	1 × 10^−7^ M	1 × 10^−6^ M
BL (C+)	31 ± 11	41 ± 4.5	70 ± 7.6
3	35 ± 3	60 ± 3 *	62 ± 12
4	45 ± 9.5 *	31 ± 5	24 ± 5.8 *
5	5 ± 2.7 *	13 ± 1.7 *	28 ± 2.5 *
6	49 ± 5 *	60 ± 5 *	78 ± 15 *
7	28 ± 3	64 ± 3 *	70 ± 10
8	24 ± 2.5	34 ± 2.5	35 ± 2.9 *
9	34 ± 2.2	45 ± 2.7	53 ± 6.3 *
3/4 (1.0:0.24)	26 ± 2.5	63 ± 2.9 *	66 ± 6.3
3/4 (1.0:0.9)	35 ± 3.8	43 ± 5.0	38 ± 5
Control (C-)	7 ± 5		

These values represent the mean ± standard deviation of two independent experiments with at least six replicates each (*n* = 12). (*) Represents experiments with a significant difference between positive control (BL) and analog treatments at the 0.05 significance level (Student’s *t*-test).

## Supplementary Materials

The following are available online at https://www.mdpi.com/1422-0067/21/5/1832/s1, Figure S1: LigPlot^+^ plots of protein ligand interactions with synthetic ligands (**3**, **4**, **6**, **7** and **8**), Figure S2: Comparison of the interactions of the heterodimer with brassinolide and synthetic ligands (**3**, **4**, **6**, **7** and **8**), Table S1: Pose analysis of docked brassinolide (**1**) and synthetic analogs (**3**, **4**, **6**, **7** and **8**), Table S2: Docked compounds–heterodimer protein contacts.

## Abbreviations

BL	Brassinolide
BRs	Brassinosteroids
CS	Castasterone
RLIT	Rice leaf inclination test
BSIB	Bean second node bioassay
SAR	Structure-activity Relationships
BRI1^LRR^	BL receptor with extracellular leucine-rich domain
BAK1	BRI1-associated kinase 1
