# Supplementary material for: Biological Activities and Molecular Docking of Brassinosteroids 24-Norcholane Type Analogs
Source: Int J Mol Sci. 2020 Mar 6;21(5):1832. doi: 10.3390/ijms21051832 (PMC7084776; doi:10.3390/ijms21051832)
Supplement: Supplementary file 1 [file ijms-21-01832-s001.docx]

Article

Biological Activities and Molecular Docking of Brassinosteroids 24-Norcholane Type Analogs

Katy Díaz ^1^, Luis Espinoza ^1^, Rodrigo Carvajal ^1^, Marcos Conde-González ^2^, Vladimir Niebla ^2^, Andrés F. Olea ^3,^* and Yamilet Coll ^2,^*

^1^ Departamento de Química, Universidad Técnica Federico Santa María, Avenida España 1680, Valparaíso 2340000, Chile; [katy.diaz@usm.cl](mailto:katy.diaz@usm.cl) (K.D.); [luis.espinozac@usm.cl](mailto:luis.espinozac@usm.cl) (L.E.); [rodrigo.carvajal@postgrado.usm.cl](mailto:rodrigo.carvajal@postgrado.usm.cl) (R.C.)

^2^ Center for Natural Products Research, Faculty of Chemistry, University of Havana. Zapata y G, La Habana 10400, Cuba; [mrconde@estudiantes.fq.uh.cu](mailto:mrconde@estudiantes.fq.uh.cu) (M.C.-G.); [vladyniem@gmail.com](mailto:vladyniem@gmail.com) (V.N.)

^3^ Instituto de Ciencias Químicas Aplicadas, Facultad de Ingeniería, Universidad Autónoma de Chile, El Llano Subercaseaux 2801, Santiago 8900000, Chile

***** Correspondence: [andres.olea@uautonoma.cl](mailto:andres.olea@uautonoma.cl) (A.F.O.); [yamcoll@fq.uh.cu](mailto:yamcoll@fq.uh.cu) (Y.C.); Tel.: +56-2-2303-6666 (A.F.O.)


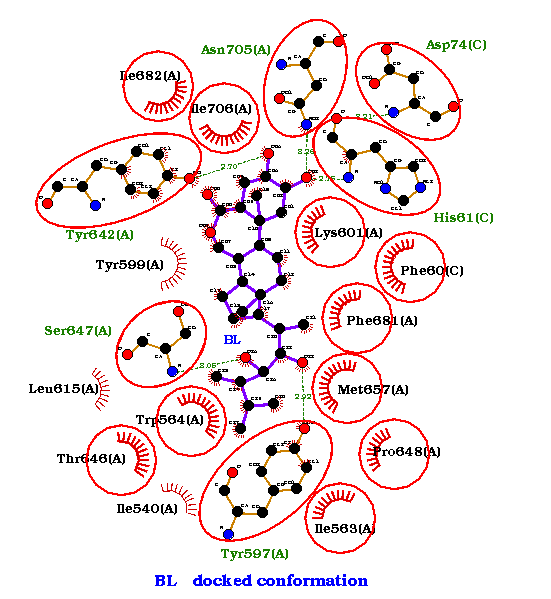


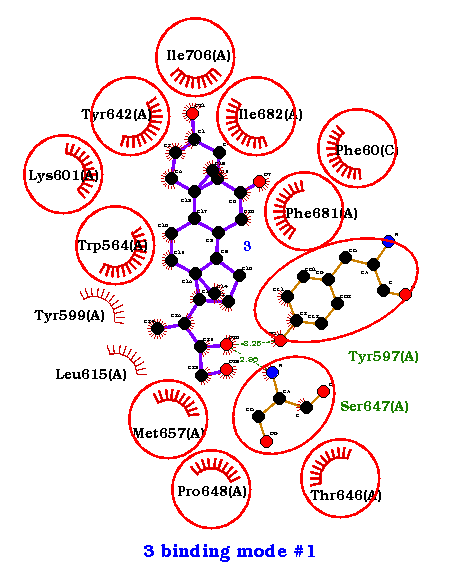

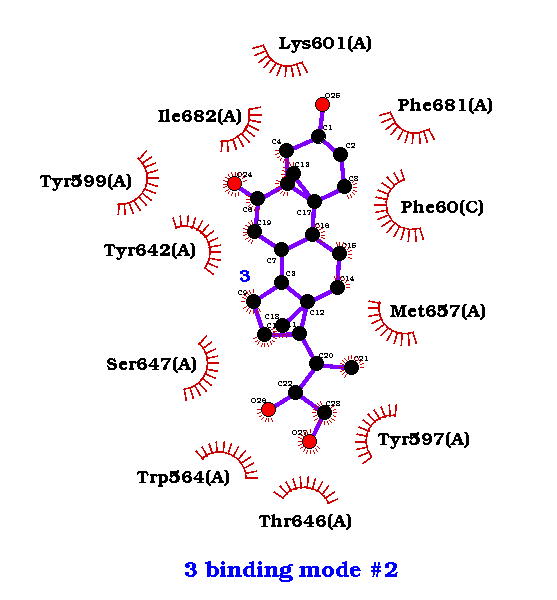


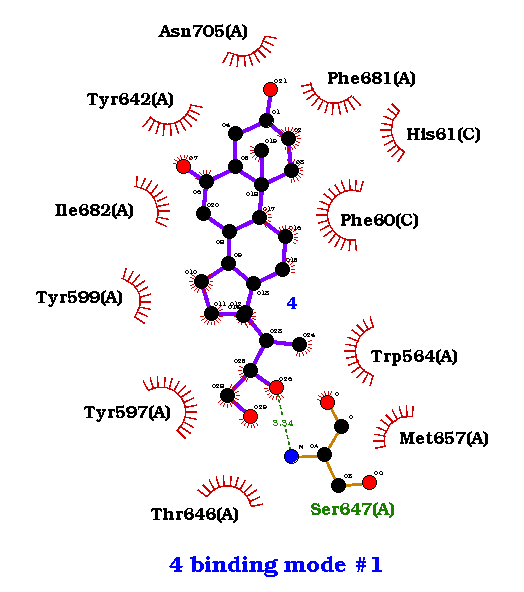


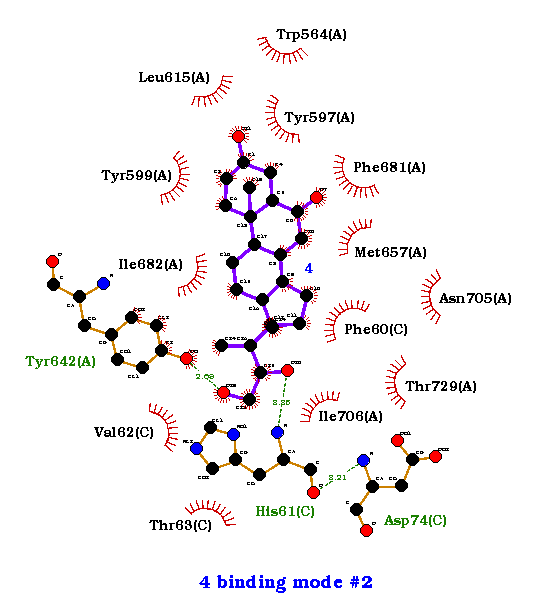


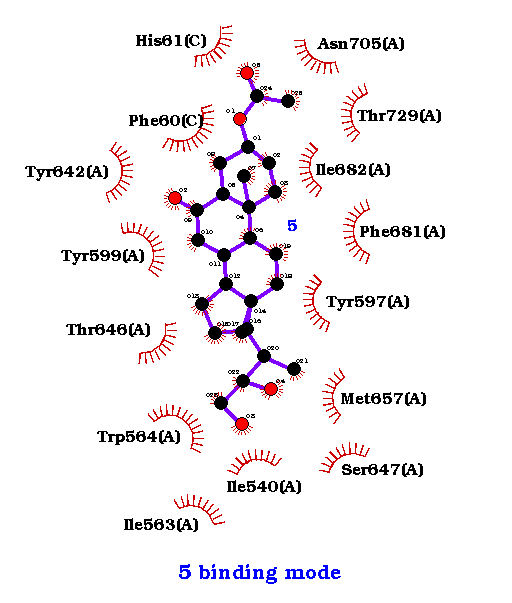


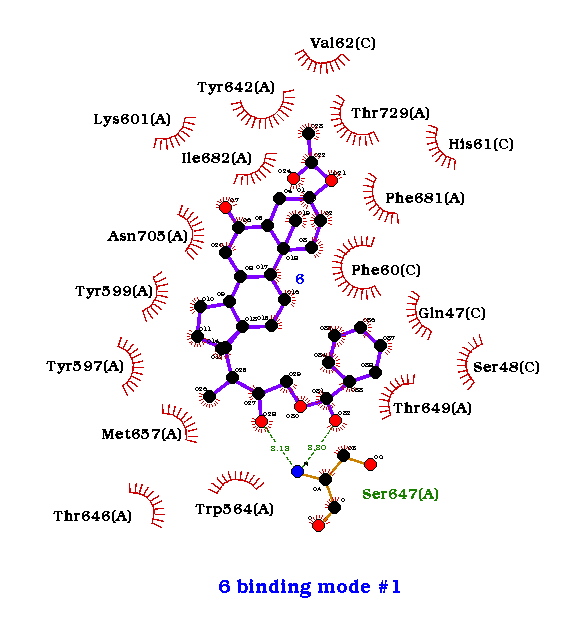


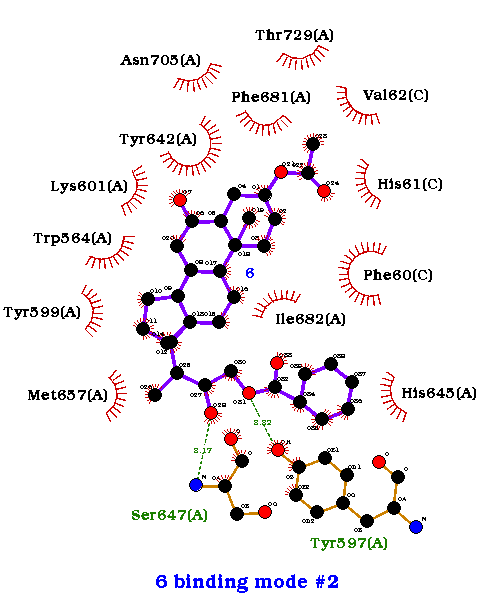


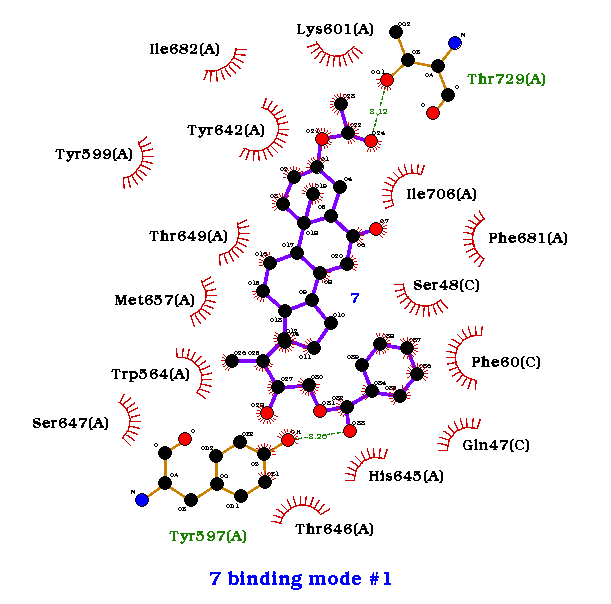


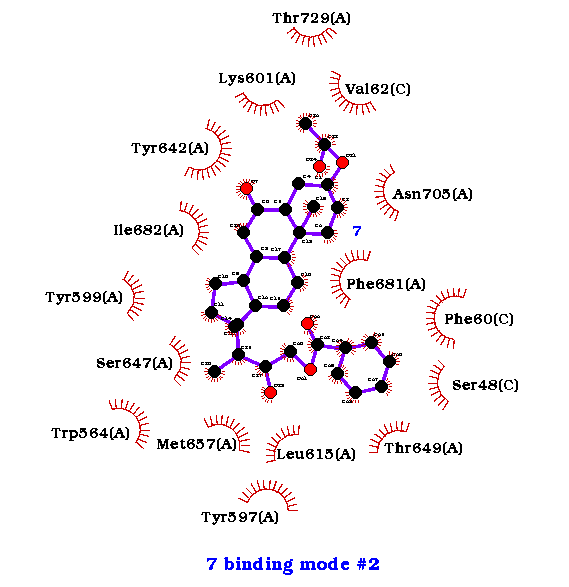


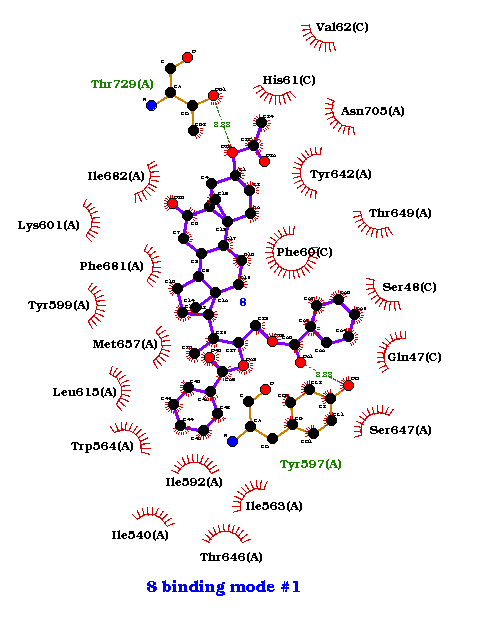


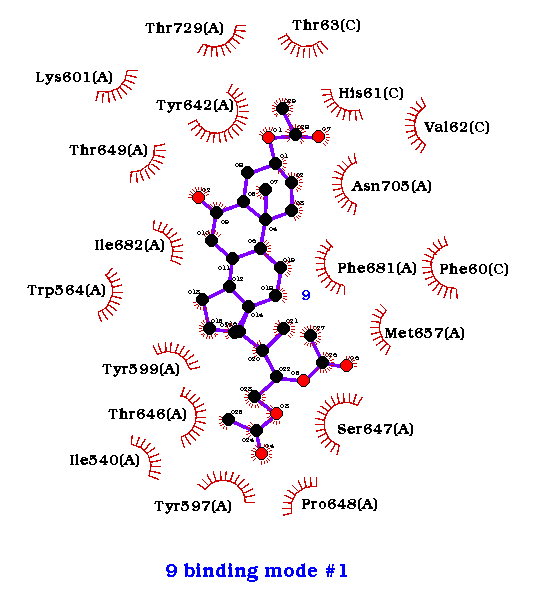


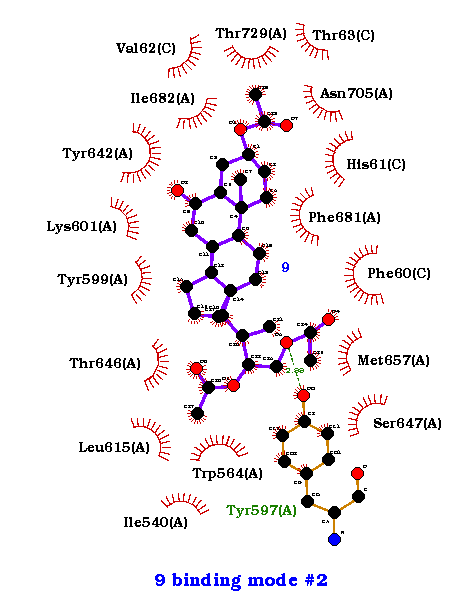


*Figure S1. LigPlot^+^ plots of protein ligand interactions with BL (****1****) and synthetic ligands: Compound* ***3****; Compound* ***4****; Compound* ***5****, Compound* ***6****, Compound* ***7****; Compound* ***8****, Compound* ***9****. BRI1 is chain A and BAK1 is chain C. Naming scheme for residues: Aaa##(chain).*

*
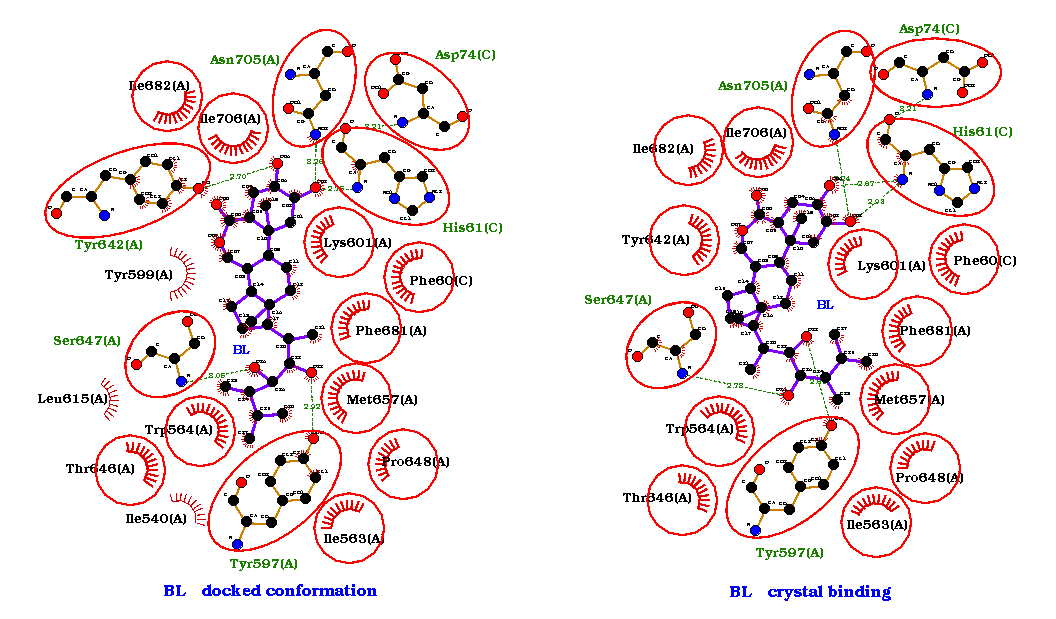
*

*
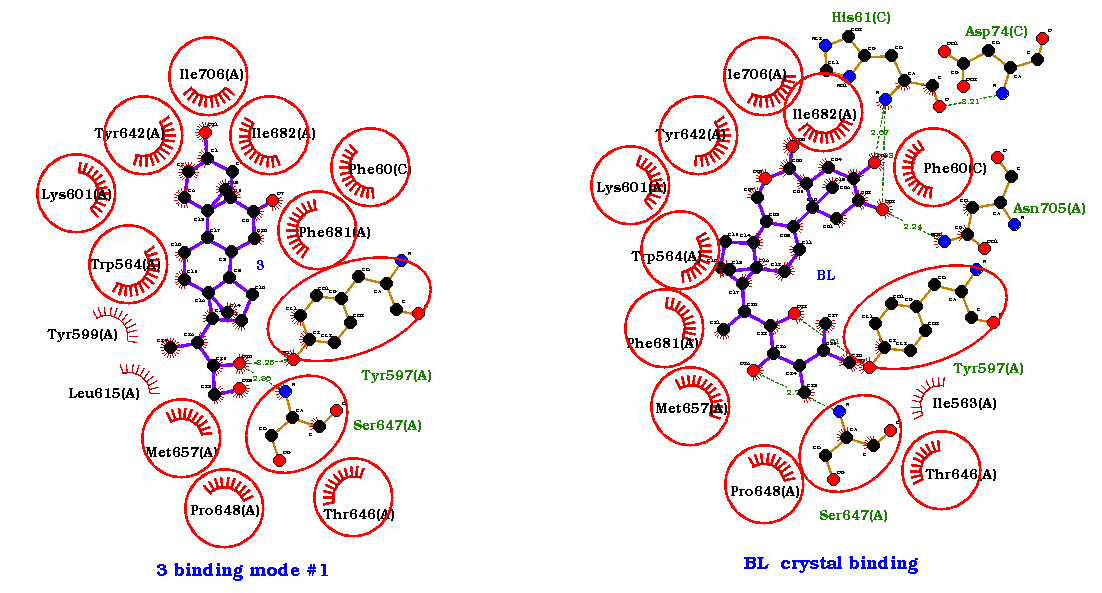
*

*
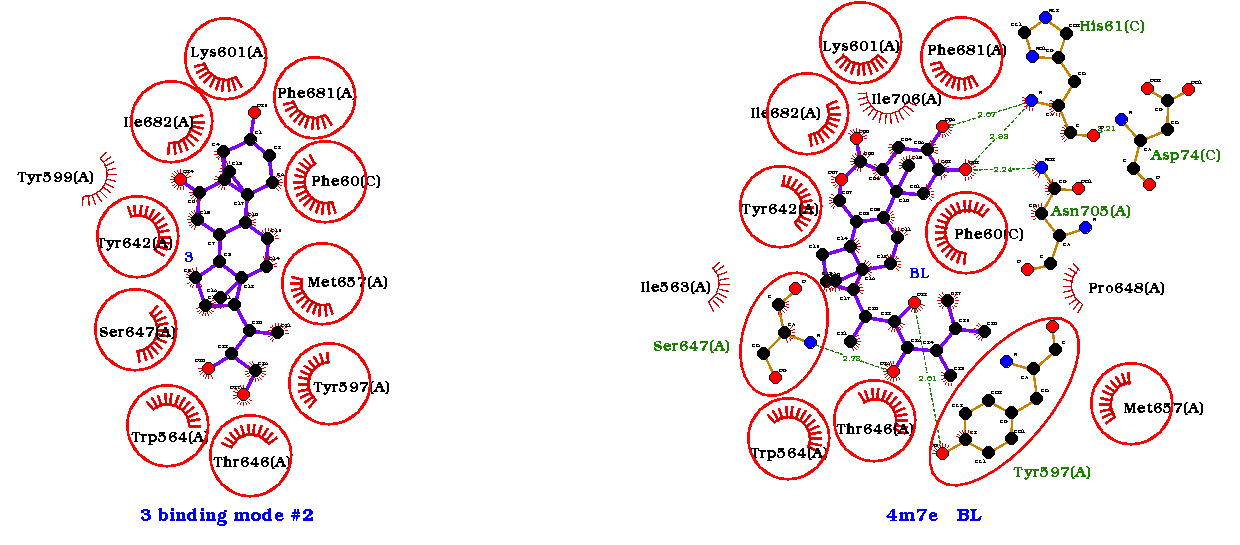
*

*
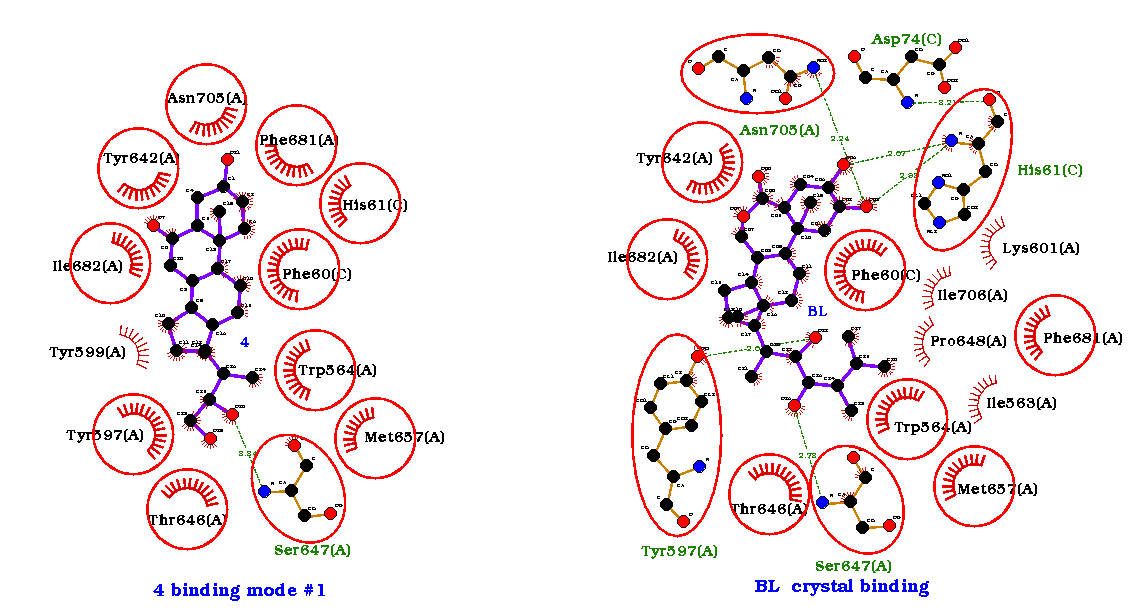
*

*
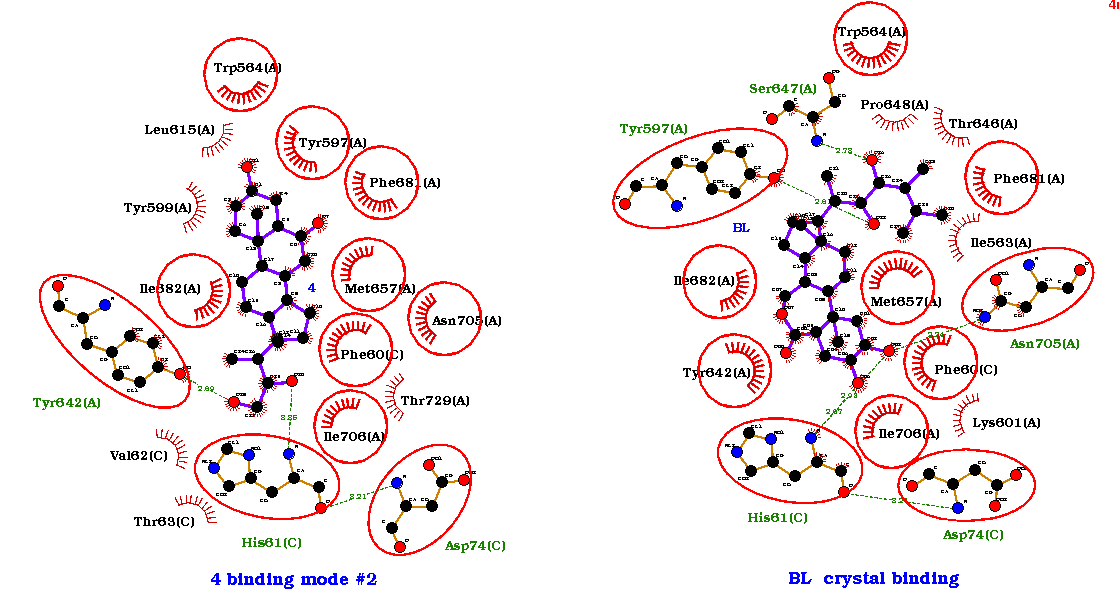
*

*
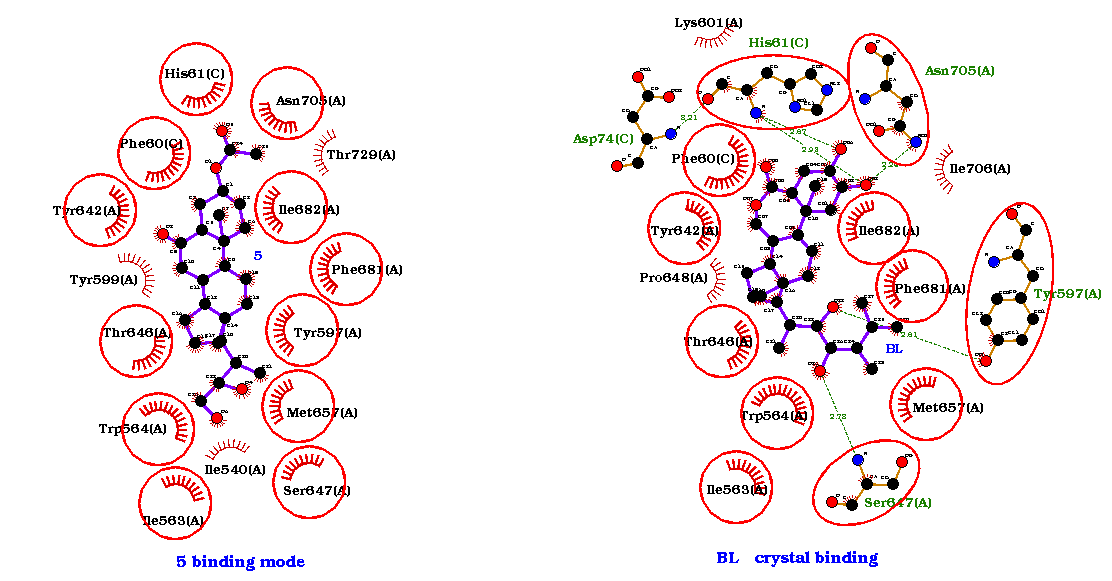
*

*
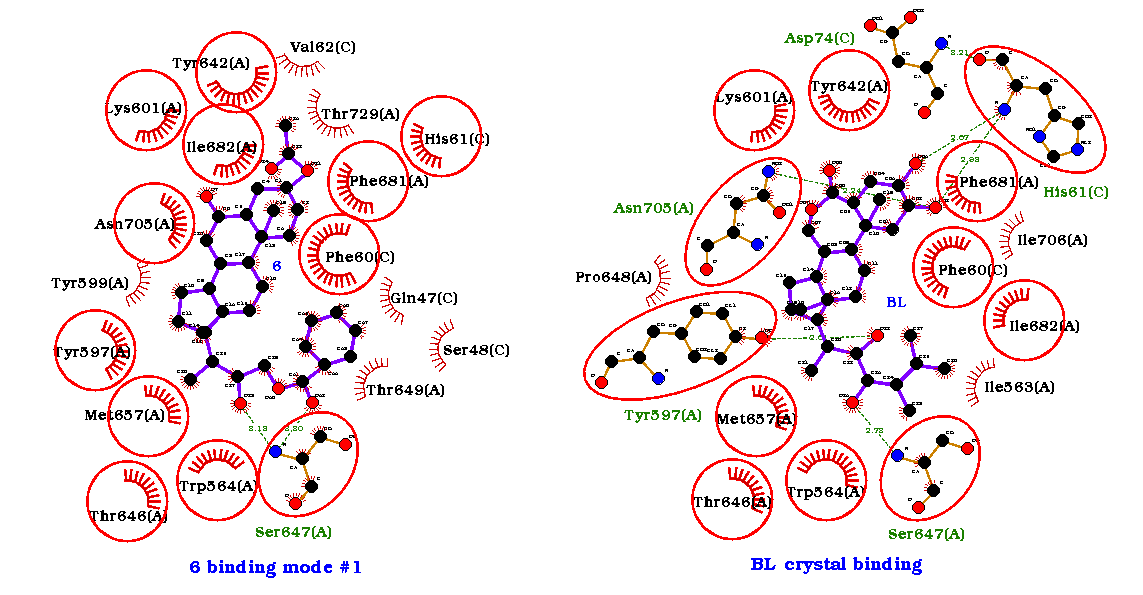
*

*
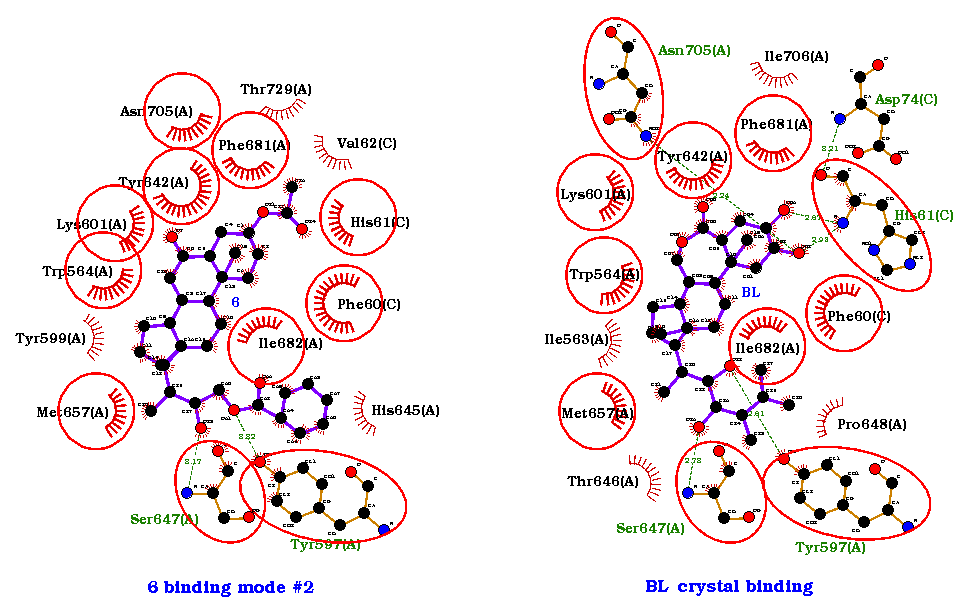
*

*
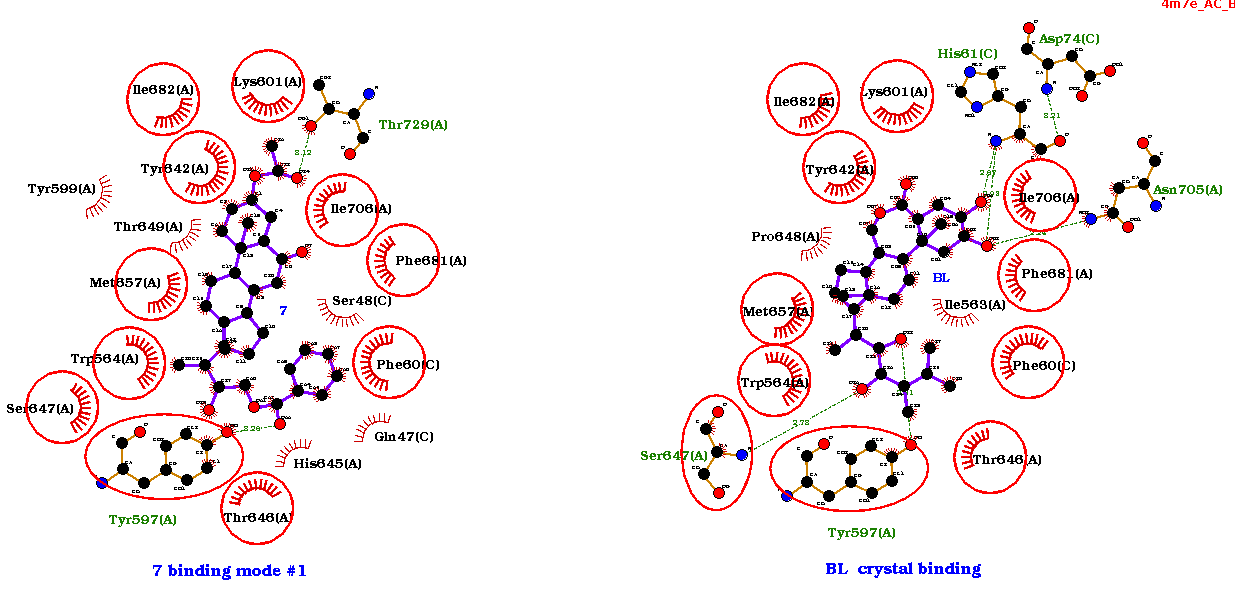
*

*
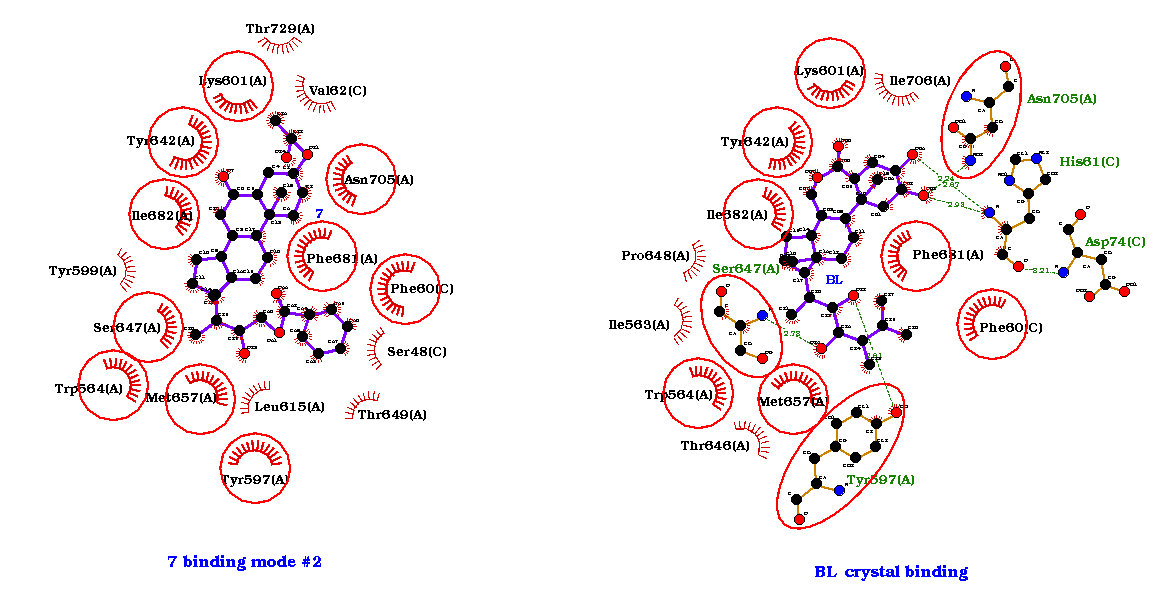
*

*
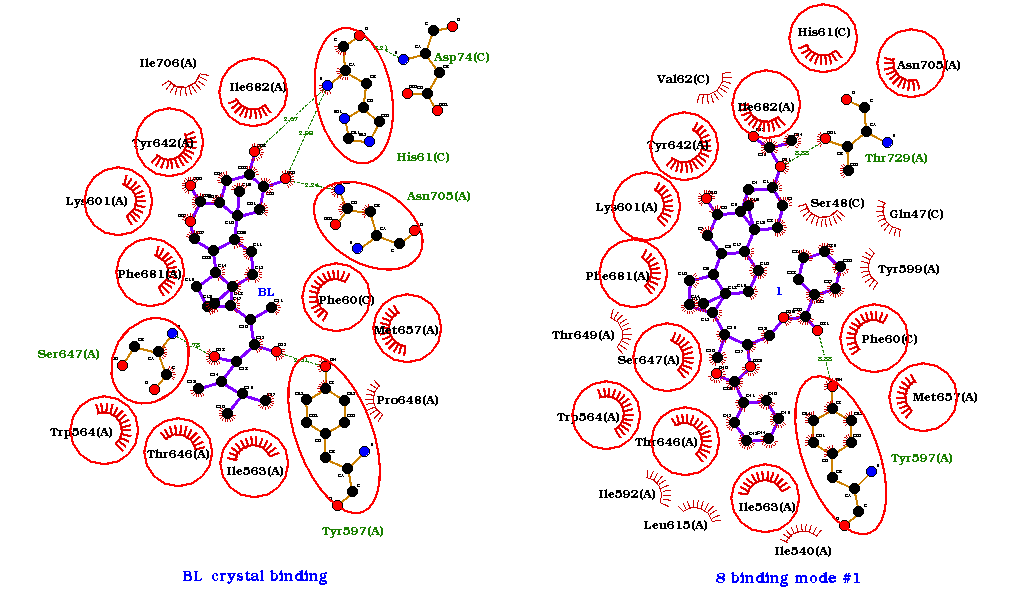
*

*Figure S2. Comparison of the interactions of the heterodimer with BL and synthetic ligands: brassinolide (****1****, BL); Compound* ***3****; Compound* ***4****; Compound* ***6****; Compound* ***7****; Compound* ***8****. (****residues encircled in red indicate common interactions with BL****). Same naming scheme as before applies.*

**Table S1.** Pose analysis of docked BL (**1**) and synthetic analogs (**3**, **4**, **6**, **7** and **8**). Num. Pos.: number of poses in cluster (RMSD < 1.5Å). ΔE_b_: Binding Energy in kcal/mol.

| Code | **Mode 1** | | **Mode 2** | | Structure |
| --- | --- | --- | --- | --- | --- |
|  | Num. Pos. | ΔE_b_ | Num. Pos. | ΔE_b_ |  |
| **1** | 15 | -12.6 | 15 | -10.8 |  |
| **3** | 15 | -11.4 | 15 | -10.8 |  |
| **4** | 15 | -11.1 | 15 | -11.0 |  |
| **6** | 16 | -12.3 | 12 | -12.1 |  |
| **7** | 15 | -11.4 | 15 | -11.2 |  |
| **8** | 19 | -12.9 | - | - |  |

**Table S2.** Docked compounds-heterodimer protein contacts. (O^s^ refers to the hydroxyl group in the side chain of Tyr CO^bz^ refers to the carbonyl oxygen in benzoate moiety, CO^Ac^ refers to the carbonyl oxygen in acetate moiety and NH_2_^s^ refers to the amide in the side chain of Asn).

| Compound | **Protein Contacts** | |
| --- | --- | --- |
|  | Hydrogen bonds | Non-polar interactions |
| **1** | O^s^Tyr642(A)-OC3  O^s^Tyr597(A)-OC22  NH_2_^S^Asn705(A)-OC2  NHHis61(C)-OC2 | Ile682(A), Ile706(A), Lys601(A), Tyr599(A), Phe681(A), Leu615(A), Met657(A), Thr646(A), Trp564(A), Pro648(A), Ile563(A), Ile540(A), Phe60(C), Asp74(C) |
| **3** (b1) | NHSer647(A)-OC22  O^s^Tyr597(A)-OC22 | Lys601(A), Ile706(A), Tyr599(A), Leu615(A), Met657(A), Trp564(A), Pro648(A), Thr649(A), Phe681(A), Tyr642(A), Ile682(A), Phe60(C) |
| **3** (b2) |  | Lys601(A), Ile682(A), Tyr599(A), Tyr642(A), Ser647(A), Trp564(A), Thr646(A), Tyr597(A), Met657(A), Phe681(A), Phe60(C) |
| **4** (b1) | NHSer647(A)-OC22 | Tyr642(A), Tyr599(A), Trp564(A), Tyr597(A), Thr646(A), Met657(A), Phe681(A), Ile682(A), Asn705(A), Phe60(C) |
| **4** (b2) | O^s^Tyr642(A)-OC23  COVal62(C)-OC22  NHHis61(C)-OC22 | Leu615(A), Trp564(A), Tyr597(A), Tyr599(A), Thr729(A), Asn705(A), Ile682(A), Ile706(A), Phe681(A), Met657(A), Phe60(C), Thr63(C) |
| **6** (b1) | NHSer647(A)-CO^bz^C23  NHSer647(A)-OC22 | Thr729(A), Asn705(A), Lys601(A), Phe681(A), Tyr642(A), Tyr599(A), Met657(A), Tyr597(A), Trp564(A), Thr649(A), Ile682(A), Val62(C), His61(C), Ser48(C), Gln(47), Phe60(C) |
| **6** (b2) | NHSer647(A)-OC22  O^s^Tyr597(A)-OC23 | Thr729(A), Asn705(A), Tyr642(A), Lys601(A), Phe681(A), Tyr599(A), Trp564(A), Met657(A), His645(A), Ile682(A), Phe60(C), His61(C), Val62(C) |
| **7** (b1) | O^s^Tyr597(A)-CO^Ac^C3  O^s^Thr729(A)-CO^bz^C23 | Lys601(A), Ile706(A), Phe681(A), Ile582(A), Tyr642(A), Met657(A), Tyr599(A), Trp564(A), Ser647(A), Thr646(A), Thr649(A), His645(A)  Gln47(C), Ser48(C), Val62(C) |
| **7** (b2) |  | Thr729(A), Lys601(A), Tyr642(A), Ile682(A), Tyr599(A), Ser647(A), Trp564(A), Met657(A), Tyr597(A), Leu615(A), Thr649(A), Phe681(A), Asn705(A), Ser48(C), Phe60(C), Val62(C) |
| **8** | O^s^Thr729(A)-OC23  O^s^Tyr597(A)-CO^bz^C23 | Asn705(A), Lys601(A), Tyr642(A), Phe681(A), Ile682(A), Met657(A), Tyr599(A), Leu615(A), Thr646(A), Ile563(A), Trp564(A), Ile540(A), Ile592(A), Ser647(A), Thr649(A), His61(C), Val62(C), Ser48(C), Gln47(C), Phe60(C) |
